# Sweet products, subtle residues: neonicotinoid contaminations in commercial honey from Thailand

**DOI:** 10.1007/s11356-026-38117-9

**Published:** 2026-08-14

**Authors:** Domenic W. Camenzind, Wankuson Chanasit, Marion Fracheboud, Christina Kast, Jakkrawut Maitip, Lars Straub

**Affiliations:** 1 Institute of Bee Health, Vetsuisse Faculty, University of Bern, Bern, Switzerland; 2 Faculty of Science and Digital Innovation, Thaksin University, Papayom, Thailand; 3 Swiss Bee Research Centre, Agroscope, Bern, Switzerland; 4 Faculty of Science, King Mongkut’s University of Technology North Bangkok, Energy, and Environment, Bankhai, Thailand

**Keywords:** Biodiversity, Pesticides, Pollinators, Pollution, Sustainability

## Abstract

Environmental pollution with pesticides, including neonicotinoid insecticides, is a major driver of biodiversity loss and raises concerns for both pollinators and human health. As a product of nectar foraging, honey integrates environmental contamination across large spatial scales, making it a valuable sentinel matrix for pesticide exposure. However, systematic data on neonicotinoid residues in commercial honey from tropical ecosystems are currently lacking. Here, we show that neonicotinoid residues are widespread in Thai commercial honey, albeit at levels posing low acute risk to humans and bees. Using ultra-high performance liquid chromatography coupled with tandem mass spectrometry (UHPLC–MS/MS; limits of quantification 0.25–1 ng g^−1^, recoveries 94–117%), residue concentrations of five commonly used neonicotinoid insecticides were quantified in 31 commercial honey samples. Neonicotinoid residues were detected in more than 60% of samples, frequently involving multiple compounds, with concentrations ranging up to 3.11 ng g^−1^. Although calculated exposure metrics indicated low acute risks for both human consumers and bees, detected residue concentrations overlapped with levels reported to induce sublethal effects in bees under chronic exposure scenarios. This first broad survey of neonicotinoid residues in Thai commercial honey demonstrates frequent low-level contamination and underscores the need for expanded pesticide monitoring that includes additional hive matrices and pollinator taxa. Such data are essential to better characterize real-world exposure scenarios and to support more sustainable pest and pollinator management in tropical agroecosystems.

**Supplementary Information:**

The online version contains supplementary material available at 10.1007/s11356-026-38117-9.

## Introduction

Since the Green Revolution, agricultural productivity has increasingly relied on synthetic pesticides, including insecticides (Ameen and Raza 2017; Devi et al. 2022). Neonicotinoid insecticides are among the most widely used globally due to their systemic properties and broad-spectrum efficacy (Jeschke et al. 2011). However, a substantial proportion of applied neonicotinoids—estimated at up to 90% (Sánchez-Bayo 2014)—fails to reach target pests and instead disperses into agricultural produce, non-crop vegetation, and the wider environment, resulting in diffuse contamination beyond agricultural landscapes (Krupke and Tooker 2020). This has raised increasing concern regarding adverse effects on pollinators and potential dietary exposure risks for humans (Klingelhöfer et al. 2022; Margaoan et al. 2024). Despite restrictions in several regions, neonicotinoids remain widely used in many countries, including Thailand, where insecticide use has increased while environmental monitoring remains limited (Dentzman et al. 2025; Nimako et al. 2025).

Honey has emerged as a valuable sentinel matrix for monitoring pesticide contamination because it integrates environmental exposure across both space and time (Perugini et al. 2011). Foraging bees collect nectar, pollen and water across large spatial scales, thereby acquiring contaminants from multiple crops, vegetation types and non-crop habitats within their foraging ranges. Within the hive, these inputs are processed and mixed, producing honey that reflects cumulative, field-realistic pesticide exposure rather than short-term contamination events (Bogdanov 2006; van der Steen 2016). Unlike point-based environmental measurements, honey captures exposure across heterogeneous landscapes and multiple flowering periods, making it well suited for assessing chronic, low-level contamination. Consistent with this integrative role, studies across diverse regions and cropping systems have demonstrated that pesticide profiles in honey mirror local pesticide use patterns and broader landscape-level contamination for water-soluble compounds, such as neonicotinoids (e.g., Han et al. 2022; Kavanagh et al. 2021; Mitchell et al. 2017; Woodcock et al. 2018). Honey therefore provides an environmentally relevant indicator linking agricultural practices, pollinator exposure, and human consumption.

Recent surveys have demonstrated that pesticide contamination of honey is a widespread phenomenon across both temperate and tropical regions. Neonicotinoid residues have been reported in honey from Europe, North America, China, Mexico, the Seychelles, and South Africa, although detection frequencies and concentrations vary considerably among regions (Adeyinka and Mketo 2025; Mubin et al. 2023; Mulati et al. 2018; Muli et al. 2018; Ponce-Vejar et al. 2022; Valdovinos-Flores et al. 2017). Despite this growing body of evidence, major geographic gaps remain. This is particularly true in Southeast Asia, where information on pesticide residues in commercially available honey remains scarce despite high pollinator diversity, year-round foraging activity, and continued pesticide use.

Thailand harbors high native bee diversity, including numerous stingless bees and several honey bee species, alongside the introduced western honey bee, *Apis mellifera* (Chuttong et al. 2014). Traditional honey hunting and beekeeping play important economic and cultural roles in Thailand (Chantawannakul et al. 2018). Honey and related products are widely consumed domestically and are also exported (Chuttong et al. 2014; Punya et al. 2024). Several native species, including *Apis cerana* and the stingless bee *Tetragonula laeviceps*, are actively managed and forage year-round. These species differ markedly in foraging range, ecology, and crop associations (Beekman and Ratnieks 2000; Smith et al. 2017). Consequently, honey produced by different bee taxa may reflect environmental pesticide contamination in complementary ways (van der Steen 2016; Wueppenhorst et al. 2024). Honey harvested from non-managed species such as *Apis dorsata* or *Apis florea* may provide complementary information on pesticide exposure across landscapes that are less influenced by managed agricultural systems, including forest habitats. Despite this potential, pesticide residue data for Thai honey remain scarce. To date, no study has systematically assessed residue levels in commercially available honey or compared exposure patterns among honey bee and stingless bee species. This knowledge gap is particularly notable because Southeast Asia remains underrepresented in the global honey residue literature despite supporting exceptionally diverse pollinator communities, extensive agricultural production, and continued intensive pesticide use (Estoque et al. 2019; Phan et al. 2024; Struebig et al. 2025; Zeng et al. 2018).

To address this gap, we analyzed commercially available honey from Thailand for five common neonicotinoid insecticides using ultra-high-performance liquid chromatography coupled with mass spectrometry (UHPLC-MS/MS). Our objectives were to (i) quantify neonicotinoid occurrence and concentrations in commercial honey, (ii) evaluate potential screening-level risks for bees and human consumers using toxicity exposure ratios and hazard indices, and (iii) generate baseline data to inform future pesticide monitoring and exposure assessments in tropical agroecosystems.

## Materials and methods

### Sample collection and classification

**Fig. 1:**
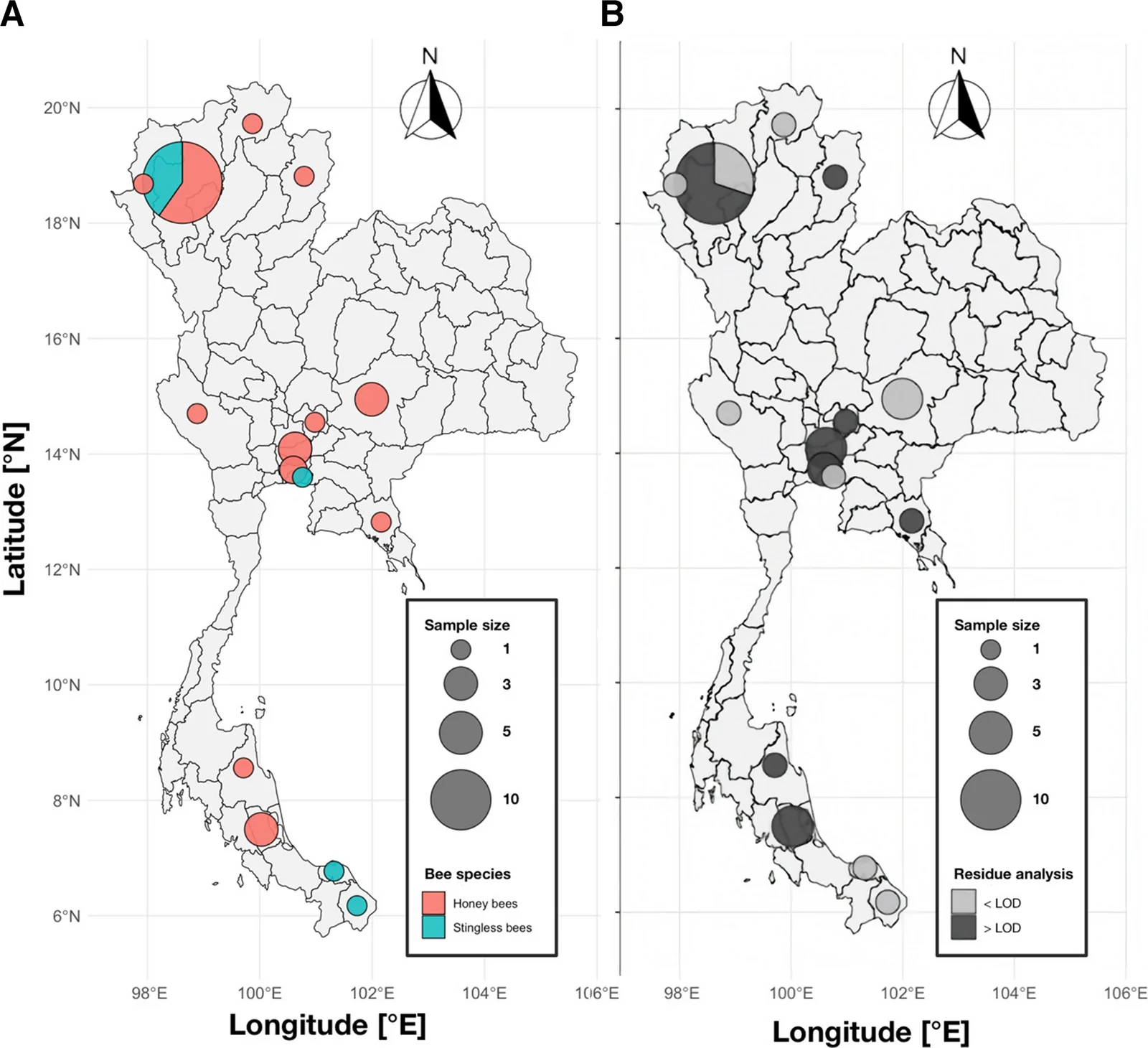
Spatial distribution of commercial honey samples collected across Thailand. Sampling locations are shown at the province level with pie chart size proportional to the number of samples collected per province. (**A**) Distribution of sample by bee origin: Red indicates samples originating from honey bee colonies, while blue represents stingless bee samples. Pie charts illustrate the relative proportion of honey- and stingless bee samples within each province. (**B**) Distribution of samples by residue detection status: Light grey indicates samples with residues below the limit of detection (LOD), while dark grey indicates samples with residues above the LOD. In total, 31 samples were collected from 15 provinces, comprising 24 honey- and seven stingless bee samples

In 2023 and 2024, a total of 31 honey samples were collected in Thailand from multiple points of sale, including beekeepers, bee hunters, and retail outlets. Bee hunters refer to collectors harvesting honey from wild bee colonies which is common throughout Thailand and Southeast Asia (Chantawannakul et al. 2018). Samples were obtained as products available to consumers at the point of sale. Samples were collected opportunistically from the consumer market and therefore reflect consumer exposure at point of sale rather than a statistically representative national monitoring program. Accordingly, the study was designed to provide a baseline assessment of neonicotinoid residues in commercially available honey across multiple regions and bee taxa in Thailand rather than to estimate national residue prevalence or geographic patterns of contamination. For subsequent analyses, samples were categorized according to purchase source as either direct purchase (i.e. honey sold by beekeepers or bee hunters at local markets or at apiaries; *N* = 21) or retail purchase (i.e., honey purchased from online platforms or physical stores; *N* = 10). For each sample, the declared province of origin was recorded and assigned to one of five geographic regions: North (*N* = 16), East (*N* = 1), West (*N* = 1), Central (*N* = 7), or South (*N* = 6) (Fig. 1A). Geographic origin was recorded as declared and may not fully reflect foraging areas in cases of honey blending. However, none of the sampled products explicitly indicated that they consisted of honey blended from multiple geographic origins, and origin information was therefore recorded according to the information provided on product labels or by vendors. Honey samples were further classified according to the bee species declared or reported on product labels. Samples originated either from honey bees (*N* = 24), including *A. mellifera*, *A. cerana*, *A. dorsata*, and *A. florea*, or from stingless bees (*N* = 7), including *T. laeviceps*, *T. pagdeni*, and *Heterotrigona itama*. Information on bee species was based on vendor declarations or product labelling and was not independently verified. Detailed information on sample origin, bee species, geographic classification, and purchase source is provided in Supplementary Information Table S1.

### Quantification of neonicotinoids

Neonicotinoids were quantified using Ultra-High Performance Liquid Chromatography-Tandem Mass Spectrometry (UHPLC-MS/MS) following a modified version of the method described for analysis of pesticides in bee bread by Schaad et al. (2023). Five neonicotinoid insecticides widely used in agricultural systems, namely thiamethoxam, clothianidin, imidacloprid, thiacloprid, and acetamiprid, were targeted. These compounds were selected because neonicotinoids remain among the most widely used systemic insecticides globally and in Thailand (Alsherbeny et al. 2026; Pesticides use and trade 2025; Wang et al. 2023), readily translocate into nectar and pollen, and are therefore particularly relevant contaminants of bee-collected resources. Furthermore, extensive toxicological data are available for both honey bees and other pollinators (Lee et al. 2016; Lewis et al. 2016), allowing residue data to be interpreted within a pollinator risk assessment framework. The selected compounds are also frequently included in international honey monitoring studies, facilitating comparison with published datasets.

The extraction of the neonicotinoids from honey was performed using a modified QuEChERS (quick, easy, cheap, efficient, rugged, safe) protocol as described in detail in the Supplementary Information Section 1.1.3 (Extraction of neonicotinoids from honey). Deuterated neonicotinoids were included as internal standards to correct for variable extraction efficiencies, potential matrix effects and detector responses.

Instrumental analysis was performed using an Agilent 1290 Infinity II coupled to an Agilent 6495 C operating in multiple reaction monitoring (MRM) mode, with a total of three ion transitions monitored per analyte. Detailed information on chromatography and mass spectrometry is provided in the supporting information in Section 1.1.4 (UHPLC-MS/MS analysis), SI Table 2 (LC gradient setting used for separation of neonicotinoid insecticides in honey), SI Table 3 (Ion source condition), SI Table 4 (Ion transitions used for qualification and quantitation) and SI Fig. 1 (Visualized linearity). Recoveries were established using blank honey samples fortified at a minimum of four concentration levels per analyte spanning the validated ranges. Recoveries were assessed in four to five replicate analyses per concentration level, yielding recoveries of 94% to 118% (SI Table 5: Recoveries at various fortification levels and repeatability). The limits of quantification (LOQ) were defined as the lowest concentration level meeting acceptable recovery rates and linearity criteria. The resulting LOQs ranged from 0.25 ng g^−1^ to 1 ng g^−1^ (SI Table 6). Limits of detection (LOD) were determined experimentally by serial dilution of spiked blank extracts until a signal-to-noise ratio of 3:1 was achieved. The LODs ranged from 0.05 ng g^−1^ to 1 ng g^−1^ depending on the neonicotinoid (SI Table 6). Values detected between the LOD and LOQ were set to the LOD for descriptive statistics, whereas values < LOD were treated as non-detects.

### Calculation of toxicity exposure ratios and hazard indices

To assess the potential risk of neonicotinoid residues to bees, toxicity exposure ratios (TERs) were calculated following guidance from European Food Safety Authority (EFSA 2013). TERs were calculated according to:

$$TER=\frac{acute\;Lethal\;Dose_{50}\left(LD_{50}\right)}{maximum\;residue\;found\;\times\;daily\;honey\;consumption}$$

where the acute LD_50_ represents the lethal dose causing 50% mortality. Maximum measured concentrations were used to estimate worst-case exposure conditions for screening-level risk characterization. Where available, species-specific acute LD_50_ values and daily honey consumption rates were used. In cases where such data was unavailable, values reported for *A. mellifera* were applied as surrogates. The use of *A. mellifera* toxicity endpoints and consumption values as surrogates for stingless bees represents a pragmatic approximation necessitated by the limited availability of species-specific data (EFSA 2023; Lewis et al. 2016). However, this approach introduces uncertainty because differences in body size, food consumption, physiology and toxicological sensitivity among bee taxa may influence actual exposure and risk (Arena and Sgolastra 2014). Consequently, TER estimates for stingless bees should be interpreted as screening-level approximations rather than species-specific risk assessments. Accordingly, lower TER values indicate higher risk, with TER values greater than 10 generally considered protective against acute lethal effects (EFSA 2013). Daily honey consumption values were derived from published literature and represent conservative estimates (EFSA 2013; Zhang et al. 2019). To estimate potential hazards associated with human consumption, hazard indices (HI) were calculated following the approach described by El-Nahhal et al. (2020). First, the mean daily intake (MDI) of neonicotinoids was estimated separately for both adults and children using:

$$MDI\;=\frac{residue\;found\;\times\;daily\;honey\;consumption}{bodyweight}$$

Daily honey consumption was assumed to be 0.8 g kg^−1^ bodyweight day^−1^, with average body weight being 15 kg for children, and 60 kg for adults, based on EFSA (EFSA 2007; Wilmart et al. 2016). These exposure estimates represent screening-level, conservative assessments and do not account for variability in individual consumption patterns. Using the MDI, the hazard quotient (HQ) was calculated as:

$$HQ=\frac{MDI}{ARfD}$$

where the acute reference dose (ARfD) represents the maximum amount of substance that can be ingested in a short period without appreciable health risk. ARfD values for individual neonicotinoids were obtained from internationally recognized databases maintained by the World Health Organization (WHO 2020). Finally, the hazard index (HI) for each honey sample was calculated as the sum of individual HQs for all detected neonicotinoids:

$$HI=\sum HQ_i$$

An HI value exceeding 1 was interpreted as indicating potential concern for human health, in line with guidance from the United States Environmental Protection Agency (US Epa et al. 2000).

### Data analysis

Given the exploratory nature of the study, the opportunistic sampling design, and the limited sample size available for several categories, statistical analyses were restricted to descriptive summaries and exploratory tests of associations. Residue occurrence and concentrations were summarized descriptively. Detection frequencies were compared among the four geographical regions, bee taxa, and purchase sources using Fisher’s exact tests, which were chosen because of the small sample size and sparse categorical data (Kim 2017). These tests were interpreted cautiously as exploratory assessments of potential associations rather than as confirmatory statistical comparisons. Analyses and figure preparation were conducted using R (R Core Team 2023) and NCSS (NCSS, LCC 2025).

## Results

### Occurrence of neonicotinoid residues and sample characteristics

**Fig. 2:**
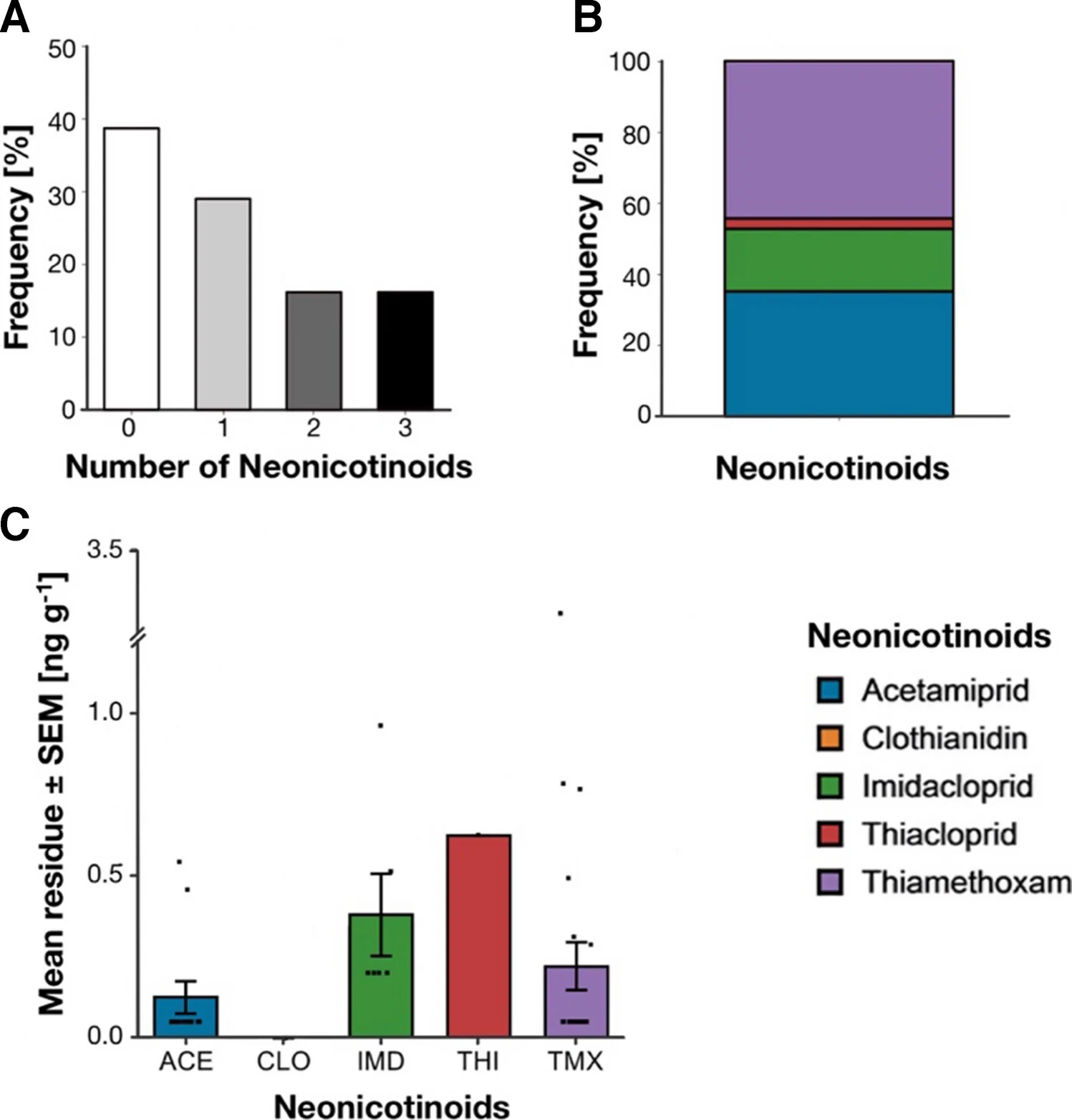
Occurrence and concentrations of neonicotinoid residues in commercial honey samples. (**A**) Relative frequency distribution [%] of the number of neonicotinoids detected above the limit of detection (LOD) per sample. Of all analysed samples, 38.71% contained no detectable neonicotinoids (white), 29.03% contained one (light grey), 16.13% contained two (dark grey), and 16.13% contained three (black) neonicotinoids above the LOD. (**B**) Relative frequency [%] of individual neonicotinoids detected above the LOD across all positive samples, shown as a stacked bar plot. Acetamiprid (blue) accounted for 35.29% of detections, imidacloprid (green) for 17.65%, thiacloprid (red) for 2.94%, and thiamethoxam (purple) for 44.12%, while clothianidin (orange) was not above the LOD in any sample. (**C**) Mean residue concentrations (ng g^−1^ ± standard error; SE) of the five analysed neonicotinoids. Where concentrations were above the LOD but below the limit of quantification (LOQ), values were set to the LOD for statistical calculations. Mean concentrations with their standard error of the mean (SEM) were 0.5 ± 0.04 ng g^−1^ for acetamiprid (blue), below the LOD for clothianidin (orange), 0.74 ± 0.22 ng g^−1^ for imidacloprid (green), 0.62 ng g^−1^ for thiacloprid (red; detected in a single sample), and 1.09 ± 0.51 ng g^−1^ for thiamethoxam (purple). All measured residues are shown as points; whiskers represent the positive and negative standard error of the mean (SEM)

Neonicotinoid residues were detected in 19 of 31 samples (61%), with six samples (19.4%) containing concentrations above the LOQ. Multiple neonicotinoid residues were detected in ten samples (31%). Of these, five samples contained two neonicotinoids, while the remaining five samples contained three compounds (Fig. 2A). Exploratory statistical analyses indicated no statistical supported association between neonicotinoid detection (i.e., above LOD) and purchase type (direct-to-consumer vs. retail), geographic region, or bee type (honey bee vs. stingless bee) (Fisher’s exact test; all *P*’s ≥ 0.4). These analyses were interpreted descriptively due to limited sample size. Detection frequencies nevertheless varied numerically among groups. By region, residues above the LOD were detected in 100% of samples from the East, 57% from central Thailand, 56% from the North 50% from the South, and 0% from the West (Fig. 2B). By bee taxon, residues were detected in 42% of Meliponini samples and 54% of Apini samples. Detection frequencies were also similar between purchase sources, with residues detected in 47% of direct-to-consumer samples and 60% of retail samples. Among the analyzed compounds, thiamethoxam was the most frequently detected neonicotinoid, occurring in 15 samples (44.1%; Fig. 2B). Acetamiprid was detected in 12 samples (35.3%), followed by imidacloprid in six samples (17.7%). Thiacloprid was detected in a single sample (2.9%) whereas clothianidin was not detected above the LOD in any sample (Fig. 2B). Detected residue concentrations were generally low. Mean concentrations (± SEM) were 1.09 ± 0.51 ng g^−1^ for thiamethoxam, 0.5 ± 0.04 ng g^−1^ for acetamiprid, and 0.74 ± 0.22 ng g^−1^ for imidacloprid. Thiacloprid was detected in one sample at 0.62 ng g^−1^ (Fig. 2C).

### TER and HI calculations

Only three honey samples yielded TER values below 100: an *A. cerana* honey containing thiamethoxam (TER = 25.66), an *A. mellifera* honey containing imidacloprid (TER = 30.11), and an *A. mellifera* honey containing thiamethoxam (TER = 50.08). All remaining samples yielded TER values greater than 100, indicating lower acute risk under the applied screening assumptions (Table 1). All calculated HIs for human consumption were ≤0.001 for both adults and children and therefore remained substantially below the threshold of concern (HI = 1) for all analyzed samples (Table 2).

**Table 1:** Toxicity exposure ratio (TER) calculations for neonicotinoid residues detected in honey produced by different bee species. TERs were calculated for samples in which neonicotinoid residues were detected, relative to the honey of the species that produced each sample. Maximum measured concentrations were combined with species-specific acute oral LD_50_ values and estimated daily sucrose consumption to calculate maximum daily uptake and TERs. Where species-specific consumption rates or toxicological endpoints were unavailable, corresponding values from *Apis mellifera* were used as surrogate data. Samples in which residues were below the limit of detection (LOD) are indicated as <LOD; corresponding TER values were not calculated and are reported as "n.a." (not applicable)

Compound	Bee species	*n *>LOD	Max. conc. (µg kg^−^^1^)	LD_50_ (µg bee^−^^1^)	Mean daily sucrose consumption (kg)	Max daily uptake (µg bee^−^^1^ day^−^^1^)	TER
Thiamethoxam	*Apis mellifera*	8	0.78	0.005	0.000128	0.00009984	50.08
	*Apis ceranae*	4	3.11	0.003	0.0000376	0.000116936	25.66
	*Apis florea*	0	<LOD	0.031	0.000128^a^	n.a.	n.a.
	*Apis dorsata*	0	<LOD	0.088	0.000128^a^	n.a.	n.a.
	*Tetragonula laeviceps*	1	0.05	0.005^a^	0.000128^a^	0.00000256	1953.13
	*Tetragonula pagdeni*	0	<LOD	0.005^a^	0.000128^a^	n.a.	n.a.
	*Heterotrigona itama*	0	<LOD	0.005^a^	0.000128^a^	n.a.	n.a.
Acetamiprid	*Apis mellifera*	7	0.54	14.53	0.000128	0.00006912	210214.12
	*Apis ceranae*	2	0.05	14.53^a^	0.00000376	0.00000188	7728723.4
	*Apis florea*	0	<LOD	14.53^a^	0.000128^a^	n.a.	n.a.
	*Apis dorsata*	0	<LOD	14.53^a^	0.000128^a^	n.a.	n.a.
	*Tetragonula laeviceps*	2	0.05	14.53^a^	0.000128^a^	0.0000000488	297745901.6
	*Tetragonula pagdeni*	0	<LOD	14.53^a^	0.000128^a^	n.a.	n.a.
	*Heterotrigona itama*	0	<LOD	14.53^a^	0.000128^a^	n.a.	n.a.
Imidacloprid	*Apis mellifera*	2	0.96	0.0037	0.000128	0.00012288	30.11
	*Apis ceranae*	3	0.2	0.0027	0.00000376	0.0000188	143.62
	*Apis florea*	0	<LOD	2.947	0.000128^a^	n.a.	n.a.
	*Apis dorsata*	0	<LOD	1.923	0.000128^a^	n.a.	n.a.
	*Tetragonula laeviceps*	0	<LOD	0.0037^a^	0.000128^a^	n.a.	n.a.
	*Tetragonula pagdeni*	0	<LOD	0.0037^a^	0.000128^a^	n.a.	n.a.
	*Heterotrigona itama*	0	<LOD	0.0037^a^	0.000128^a^	n.a.	n.a.
Thiacloprid	*Apis mellifera*	1	0.62	17.32	0.000128	0.000079616	217544.21
	*Apis ceranae*	0	<LOD	17.32^a^	0.00000376	n.a.	n.a.
	*Apis florea*	0	<LOD	17.32^a^	0.000128^a^	n.a.	n.a.
	*Apis dorsata*	0	<LOD	17.32^a^	0.000128^a^	n.a.	n.a.
	*Tetragonula laeviceps*	0	<LOD	17.32^a^	0.000128^a^	n.a.	n.a.
	*Tetragonula pagdeni*	0	<LOD	17.32^a^	0.000128^a^	n.a.	n.a.
	*Heterotrigona itama*	0	<LOD	17.32^a^	0.000128^a^	n.a.	n.a.
Clothianidin	*Apis mellifera*	0	<LOD	0.004	0.000128	n.a.	n.a.
	*Apis ceranae*	0	<LOD	0.0005	0.00000376	n.a.	n.a.
	*Apis florea*	0	<LOD	0.036	0.000128^a^	n.a.	n.a.
	*Apis dorsata*	0	<LOD	0.22	0.000128^a^	n.a.	n.a.
	*Tetragonula laeviceps*	0	<LOD	0.004^a^	0.000128^a^	n.a.	n.a.
	*Tetragonula pagdeni*	0	<LOD	0.004^a^	0.000128^a^	n.a.	n.a.
	*Heterotrigona itama*	0	<LOD	0.004^a^	0.000128^a^	n.a.	n.a.

^a^Values derived from *A. mellifera*

**Table 2:** Human Hazard quotient (HQ) and hazard index (HI) calculations of positive samples for both adults and children. Hazard quotients (HQ) were calculated each individual sample based on measured concentrations and compound specific acute reference doses (ARfD). Hazard indices (HI) were derived as the sum of HQs for samples containing multiple residues and were calculated separately for adults and children. For samples containing a single neonicotinoid residue, the HQ is equal to the HI. Only samples with residues detected above the limit of detection are shown

Sample	Species	Neonicotinoid			MDI (ng g^−^^1^)		HQ	
		Name	Conc (ng g^−^^1^)	ARfD	Adult	Child	Adult	Child
2	*A. mellifera*	Thiacloprid	0.62	30	0.008	0.033	<0.001	0.001
3	*T. laeviceps*	Thiamethoxam	0.05	1000	0.001	0.003	<0.001	<0.001
5	*A. cerana*	Thiamethoxam	3.11	1000	0.041	0.166	<0.001	<0.001
		Imidacloprid	0.2	50	0.003	0.011	<0.001	<0.001
		Acetamiprid	0.05	100	0.001	0.003	<0.001	<0.001
	HI						<0.001	<0.001
7	*A. mellifera*	Thiamethoxam	0.05	1000	0.001	0.003	<0.001	<0.001
8	*A. mellifera*	Thiamethoxam	0.05	1000	0.001	0.003	<0.001	<0.001
		Acetamiprid	0.05	100	0.001	0.003	<0.001	<0.001
	HI						<0.001	<0.001
9	*A. mellifera*	Thiamethoxam	0.78	1000	0.010	0.042	<0.001	<0.001
		Acetamiprid	0.05	100	0.001	0.003	<0.001	<0.001
	HI						<0.001	<0.001
10	*A. cerana*	Thiamethoxam	0.12	1000	0.002	0.006	<0.001	<0.001
11	*A. mellifera*	Thiamethoxam	0.05	1000	0.001	0.003	<0.001	<0.001
12	*A. mellifera*	Thiamethoxam	0.05	1000	0.001	0.003	<0.001	<0.001
		Imidacloprid	0.2	50	0.003	0.011	<0.001	<0.001
		Acetamiprid	0.05	100	0.001	0.003	<0.001	<0.001
	HI						<0.001	<0.001
13	*A. mellifera*	Thiamethoxam	0.05	1000	0.001	0.003	<0.001	<0.001
		Acetamiprid	0.05	100	0.001	0.003	<0.001	<0.001
	HI						<0.001	<0.001
15	*A. cerana*	Thiamethoxam	0.05	1000	0.001	0.003	<0.001	<0.001
		Imidacloprid	0.2	50	0.003	0.011	<0.001	<0.001
	HI						<0.001	<0.001
16	*A. mellifera*	Thiamethoxam	0.49	1000	0.007	0.026	<0.001	<0.001
		Imidacloprid	0.51	50	0.007	0.027	<0.001	<0.001
		Acetamiprid	0.54	100	0.007	0.029	<0.001	<0.001
	HI						<0.001	<0.001
17	*A. mellifera*	Thiamethoxam	0.11	1000	0.001	0.006	<0.001	<0.001
		Acetamiprid	0.05	100	0.001	0.003	<0.001	<0.001
	HI						<0.001	<0.001
18	*A. mellifera*	Thiamethoxam	0.76	1000	0.010	0.041	<0.001	<0.001
		Imidacloprid	0.96	50	0.013	0.051	<0.001	0.001
		Acetamiprid	0.05	100	0.001	0.003	<0.001	<0.001
	HI						<0.001	0.001
20	*H. itama*	Acetamiprid	0.05	100	0.001	0.003	<0.001	<0.001
23	*A. cerana*	Thiamethoxam	0.29	1000	0.004	0.015	<0.001	<0.001
		Imidacloprid	0.2	50	0.003	0.011	<0.001	<0.001
		Acetamiprid	0.05	100	0.001	0.003	<0.001	<0.001
	HI						<0.001	<0.001
24	*T. laeviceps*	Acetamiprid	0.05	100	0.001	0.003	<0.001	<0.001
25	*T. laeviceps*	Acetamiprid	0.05	100	0.001	0.003	<0.001	<0.001
26	*A. cerana*	Acetamiprid	0.05	100	0.001	0.003	<0.001	<0.001

## Discussion

Our data show that more than 60% of the commercial honey samples from Thailand contained residues of at least one neonicotinoid insecticide, albeit at low concentrations (0.3 ± 0.56 ng g^−1^). This frequent detection, including the co-occurrence of multiple active substances in several samples, underscores the widespread use and environmental persistence of neonicotinoids across Thailand’s landscape. While estimated risks to human health were negligible, detected residue levels may be relevant for bees under chronic exposure scenarios, particularly given repeated low-dose intake and mixture exposure (El-Nahhal 2020; Stanley and Preetha 2016; Thompson 2016). Within Thailand’s heterogeneous landscapes and diverse pollinator communities, our findings suggest that reliance on commercial honey alone may underestimate ecologically relevant exposure scenarios, underscoring the need for monitoring strategies that more efficiently inform pollinator risk assessment and pesticide management.

The detection frequency observed here (61%) is broadly consistent with international honey surveys. A global assessment reported neonicotinoid residues in 75% of honey samples worldwide, with 45% containing multiple compounds (Mitchell et al. 2017), and similar patterns have been reported across Europe and Asia (e.g., Kavanagh et al. 2021; Wang et al. 2022; Woodcock et al. 2018). In contrast, substantially higher frequencies (up to 98%) have been revealed in regions with intensive neonicotinoid use, such as China (Han et al. 2022). Comparable patterns have also been reported from other tropical regions, although available data remain geographically fragmented. In Mexico, Valdovinos-Flores et al. (2017) detected imidacloprid in approximately 60% of honey samples, while Ponce-Vejar et al. (2022) reported pesticide residues in 63% of samples, with neonicotinoids among the most frequently detected compounds. Similarly, Muli et al. (2018) found widespread pesticide contamination in honey from the Seychelles Archipelago, including the occurrence of imidacloprid residues, whereas recent monitoring in South Africa revealed frequent detections of multiple neonicotinoids (Adeyinka and Mketo 2025). In contrast, Mubin et al. (2023) reported no detectable imidacloprid residues in selected honey samples from Indonesia. Together, these studies suggest that low-level pesticide contamination of honey is common across many tropical agroecosystems, although residue occurrence and concentrations vary considerably among regions.

Such variation is likely driven by a combination of differences in pesticide use intensity and crop composition, landscape structure, the proportion of agricultural versus natural habitats within bee foraging ranges, and methodological differences among studies, including in analytical sensitivity (e.g., LOD), target compounds, and sampling design, crop (Jones and Turnbull 2016; Ponce-Vejar et al. 2022; Woodcock et al. 2018). Furthermore, regional differences in pollinator species, foraging behaviour, and seasonality may influence the extent to which honey integrates environmental contamination. The present study was not designed to evaluate geographic or market-specific differences in residue occurrence. The opportunistic sampling design and uneven regional representation were intended to capture the diversity of honey products available to consumers rather than provide statistically representative coverage of Thailand’s honey-producing regions. Consequently, detection frequencies should be interpreted as baseline evidence of widespread low-level contamination rather than national prevalence estimates. Honey represents an integrative exposure matrix, reflecting foraging across heterogeneous landscapes, and the sampling design did not allow reconstruction of land use within the foraging ranges, a key determinant of pesticide exposure (Kavanagh et al. 2021; Ponce-Vejar et al. 2022; Schaad et al. 2023). Nevertheless, the compounds most frequently detected here—thiamethoxam (48%), acetamiprid (38%) and imidacloprid (16%)—mirror national import data reported by the Thailand pesticide alert network (Thai 2024) and are consistent with residue patterns reported in Thai agricultural products, and human biomonitoring studies (Nimako et al. 2025; Suwannarin et al. 2020). The concordance across environmental, food and human exposure data supports the interpretation that the residues detected in honey reflect field-realistic, landscape-scale contamination.

Residue concentrations ranged from 0.05 to 3.11 ng g^−1^, remaining well below levels reported from intensively farmed regions in China and Ireland (18–42 ng g^−1^; (Han et al. 2022; Kavanagh et al. 2021). Accordingly, calculated TER and HI values indicate that acute risks to consumers and bees are unlikely under the conservative screening assumptions applied here. Importantly, however, these assessments are based on acute toxicity endpoints (LD_50_ values) and therefore primarily address the likelihood of mortality rather than sublethal or chronic effects. Consequently, high TER values should not be interpreted as evidence that detected residues are biologically inconsequential. At the maximum concentration detected, a foraging honey bee would ingest approximately 0.124 ng day⁻^1^, corresponding to 37% below chronic exposure trigger thresholds (EFSA, 2018). Experimental studies demonstrated that chronic exposure at similar or lower concentrations can induce sublethal effects, including impaired learning, homing, foraging efficiency, flight and reproductive physiology and survival (Christen et al. 2024, 2016; Henry et al. 2012; Strobl et al. 2021). Furthermore, acute toxicity data remain unavailable for many tropical pollinator species, including most stingless bees. As a result, the use of *A. mellifera* toxicity endpoints likely introduces additional uncertainty, particularly given growing evidence that pesticide sensitivity can differ substantially among insect species (Arena and Sgolastra 2014). Indeed, smaller body size and higher mass-specific food requirements characteristic of many stingless bees suggest that honey bee-derived TER values may underestimate exposure and therefore underestimate risk for some species (Gradish et al. 2019). Therefore, the low risk identified by the TER analysis should be interpreted as a conservative screening outcome rather than as evidence for the absence of ecological effects. Lastly, given that bees are exposed to multiple neonicotinoids across space and time, additive or synergistic mixture effects may further amplify sublethal impacts at individual and colony level (e.g., Siviter et al. 2021).

Our data revealed no evidence that neonicotinoid occurrence in honey varied with bee life-history traits such as foraging range. Although landscape-scale studies suggest that wide-ranging foragers, including *A. mellifera,* may experience higher pesticide exposure than species with more restricted foraging ranges (Rollin et al. 2013; Knapp et al. 2023), such differences could not be resolved here. Beyond foraging range, the natural migratory behavior of native Asian honey bee species represents an additional and largely overlooked exposure pathway. Species such as *A. cerana*, *A. florea* and *A. dorsata* undertake migrations from tens to over hundreds of kilometers (Aryal 2019; Hepburn and Radloff 2011; Oldroyd and Wongsiri 2006), foraging across various agricultural and natural habitats (Robinson 2012; Sihag 2014; Traynor et al. 2016). Consequently, migratory species may experience pesticide exposure across a broader range of landscapes than non-migratory species, potentially influencing cumulative exposure over time. In this study, no neonicotinoid residues were detected in honey collected by bee hunters from *A. dorsata* and *A. florea* colonies. These species are not managed and typically nest outside intensively cultivated agricultural landscapes, often in forested or urban environments (Sihag 2017; Srinivasan et al. 2020). However, the absence of detectable residues in these samples should not be interpreted as an absence of an possible exposure risk, as neonicotinoid contaminate natural and urban habitats by drift, runoff, or atmospheric deposition (Bonmatin et al. 2015; Krupke and Tooker 2020), raising concerns that neonicotinoids pose risks to wild pollinators and broader biodiversity across multiple habitat types (Ong et al. 2025). However, empirical data remain scarce (Oldroyd and Nanork 2009); this highlights the need to explicitly consider migratory behavior when assessing pesticide risks to native Asian pollinators.

In contrast, stingless bees—key pollinators in tropical systems (Meléndez Ramírez et al. 2018; Putra, Permana, and Kinasih 2014)—typically forage over smaller spatial ranges (Beekman and Ratnieks 2000; Smith et al. 2017), potentially confining exposure to local contamination sources. In addition, stingless bee species, often of a smaller body size than honey bees experience reduced internal dilution when ingesting the same concentration of a compound, resulting in a higher internal concentration per unit of body mass (Jütte et al. 2023), while elevated mass-specific metabolic rates necessitate a higher relative food intake, potentially increasing pesticide exposure per individual (Darveau et al. 2005; Sgolastra et al. 2019). Furthermore, stingless bee larvae are mass-provisioned and consume a fixed food mass continuously over an extended developmental period, in contrast to the progressive feeding of honey bee larvae, thereby increasing both exposure duration and the effective cumulative dose during larval development (Cham et al. 2019). Together, these differences indicate that pesticide exposure and susceptibility may vary substantially among pollinator taxa in Thailand, reinforcing the need for integrative exposure assessments beyond single species or matrices.

Several limitations should be considered when interpreting our findings. First, the present study focused exclusively on five neonicotinoid insecticides and therefore does not capture the full spectrum of pesticide exposure experienced by pollinators and consumers. Other pesticide classes, including fungicides, pyrethroids, organophosphates and herbicides, are widely used in agricultural systems and may also occur in bee-collected resources. Second, pesticide exposure was assessed exclusively through honey. Although honey is a valuable sentinel matrix that integrates environmental contamination across landscapes and time, it captures only part of the exposure experienced by pollinators. In particular, pollen often contains higher pesticide concentrations and a greater diversity of compounds than honey and represents a primary route through which contaminants are transferred to developing brood. This distinction is particularly relevant because honey primarily reflects nectar-derived exposure, whereas pollen constitutes the principal protein source for larvae and may therefore better represent pesticide exposure during sensitive developmental stages (Friedli et al. 2020). Similarly, wax can accumulate pesticides over extended periods and act as a chronic source of exposure within colonies, while analyses of bee tissue provide direct information on realized internal exposure (Schott et al. 2017; Zhang et al. 2021). Future monitoring efforts would therefore benefit from integrated multi-residue and multi-matrix approaches combining honey with pollen, wax and bee samples to provide a more comprehensive assessment of pesticide exposure in tropical pollinator systems.

## Conclusion

This study provides the first systematic assessment of neonicotinoid residues in commercially available honey from Thailand and demonstrates that low-level contamination is widespread across products derived from diverse bee taxa and regions. Although detected concentrations were unlikely to pose acute risks to human consumers or bees under current screening frameworks, the frequent co-occurrence of neonicotinoids and their overlap with concentrations associated with documented sublethal effects in bees highlight important limitations of assessments based solely on lethal toxicity endpoints. Our findings therefore confirm that honey is a valuable sentinel matrix for detecting landscape-scale pesticide contamination in tropical agroecosystems, while also illustrating that honey alone captures only part of the exposure experienced by pollinators. More broadly, this study contributes to a growing body of evidence that pesticide contamination of honey occurs across tropical agroecosystems and highlights the need for expanded monitoring throughout Southeast Asia. Finally, advancing species-specific exposure and toxicity data—particularly for stingless bees and other *Apis* species—will be essential for developing pollinator risk assessments that better reflect the ecological diversity and exposure realities of tropical agricultural systems (Chuttong et al. 2014; Pibul and Jawjit 2025). Such information can support evidence-based pesticide stewardship, targeted monitoring programs, and the development of pollinator protection strategies tailored to tropical agricultural landscapes (Dicks et al. 2021; Potts et al. 2016).

## Supplementary Information

Below is the link to the electronic supplementary material.

ESM 1 (DOCX 364 KB)

## Data Availability

The complete raw data and code used for analysis can be found here: 10.5061/dryad.tdz08kqd5.
