# Supplementary material for: Sweet products, subtle residues: neonicotinoid contaminations in commercial honey from Thailand
Source: Environ Sci Pollut Res Int. 2026 Aug 14;33(26):13489–501. doi: 10.1007/s11356-026-38117-9 (PMC13503417; doi:10.1007/s11356-026-38117-9)
Supplement: Supplementary file 1 [file 11356_2026_38117_MOESM1_ESM.docx]

**Title:** Sweet products, subtle residues: Neonicotinoid contaminations in commercial honey from Thailand

**Authors:** Domenic W. Camenzind^1*^, Wankuson Chanasit^2^, Marion Fracheboud^3^, Christina Kast^3^, Jakkrawut Maitip^4^, Lars Straub^1,4^

**Affiliations:**

^1^ Institute of Bee Health, Vetsuisse Faculty, University of Bern, Bern, Switzerland^2^

^2^Faculty of Science and Digital Innovation, Thaksin University, Papayom, Thailand

^3^ Swiss Bee Research Centre, Agroscope, Bern, Switzerland

^4^ Faculty of Science, Energy, and Environment, King Mongkut’s University of Technology North Bangkok, Bankhai, Thailand

***Corresponding Author:** Domenic W. Camenzind: [domenic.camenzind@unibe.ch](mailto:domenic.camenzind@unibe.ch)

**Key words**: biodiversity, pesticides, pollinators, pollution, sustainability

**Abstract:**

Environmental pollution with pesticides, including neonicotinoid insecticides, is a major driver of biodiversity loss and raises concerns for both pollinators and human health. As a product of nectar and honeydew foraging, honey integrates environmental contamination across large spatial scales, making it a valuable sentinel matrix for pesticide exposure. However, systematic data on neonicotinoid residues in commercial honey from tropical ecosystems are currently lacking. Here, we show that neonicotinoid residues are widespread in Thai commercial honey, albeit at levels posing low acute risk to humans and bees. Using ultra-high performance liquid chromatography coupled with tandem mass spectrometry (UHPLC–MS/MS; limits of quantification 0.25–1 ng g^-1^, recoveries 94–117%), residue concentrations of five commonly used neonicotinoid insecticides were quantified in 31 commercial honey samples. Neonicotinoid residues were detected in more than 60% of samples, frequently involving multiple compounds, with concentrations ranging up to 3.11 ng g^-1^. Although calculated exposure metrics indicated low acute risks for both human consumers and bees, detected residue concentrations overlapped with levels reported to induce sublethal effects in bees under chronic exposure scenarios. This first broad survey of neonicotinoid residues in Thai commercial honey demonstrates frequent low-level contamination and underscores the need for expanded pesticide monitoring that includes additional hive matrices and pollinator taxa. Such data are essential to better characterize real-world exposure scenarios and to support more sustainable pest and pollinator management in tropical agroecosystems.

**SI Table 1:** **Sample specifications and classification:** For each honey sample, the producing bee species was recorded and classified by bee type. The province of origin was documented and assigned to one of five geographic regions within Thailand. Samples were further categorised by sales method, distinguishing direct sales from those obtained via retail outlets.

**SI Table 2.** **LC gradient setting**

|  | Time [min] | mpA | mpB |
| --- | --- | --- | --- |
| 1 | 0.25 | 100% | 0% |
| 2 | 3.50 | 60% | 40% |
| 3 | 4.50 | 0% | 100% |
| 4 | 6.50 | 0% | 100% |

Mobile phase A (mpA): 95% water + 5% acetonitrile + 0.01% formic acid + 5 mM ammonium formate; Mobile phase B (mpB): 5% water + 95% acetonitrile + 0.01% formic acid + 5 mM ammonium formate.

| **Ion source conditions** |  |
| --- | --- |
| Gas Temperature [°C] | 160 |
| Gas flow [l/ min] | 19 |
| Sheath gas temp [°C] | 400 |
| Sheath gas flow [l/ min] | 12 |
| Nebulizer [psi] | 35 |
| Capillary positive [V] | 3’000 |
| Capillary negative [V] | - |
| Nozzle voltage positive [V] | 0 |
| Nozzle voltage negative [V] | - |

**SI Table 3. Ion source conditions**

| **SI Table 4.** **Ion transitions for qualification and quantitation** | | | | | | | | | | | |
| --- | --- | --- | --- | --- | --- | --- | --- | --- | --- | --- | --- |
| Analyte |  | Precursor ion (m/z) | Quantifier | | Qualifier 1 | | | Qualifier 2 | | |  |
|  | |  | Product ion | CE^a^ (V) | Product ion | | CE^a^ (V) | Product ion | | CE^a^ (V) |  |
|  | |  |  |  |  |  | |  |  | |  |
| Acetamiprid |  | 223.1 | 126.0 | 22 | 99.0 | 46 | | 56.1 | 14 | |  |
| Acetamiprid-D3 (IS) |  | 226.0 | 126.0 | 22 | 73.0 | 66 | | - | - | |  |
| Clothianidin |  | 250.0 | 131.9 | 18 | 110.0 | 30 | | 113.0 | 30 | |  |
| Clothianidin-D3 (IS) | | 253.0 | 131.9 | 18 | 113.0 | 34 | | 45.1 | 66 | |  |
| Imidacloprid |  | 256.1 | 209.0 | 14 | 175.1 | 18 | | 84.0 | 22 | |  |
| Imidacloprid-D4 (IS) |  | 260.1 | 213.1 | 18 | 179.1 | 22 | | - | - | |  |
| Thiacloprid |  | 253.0 | 126.1 | 22 | 90.2 | 46 | | 73.1 | 78 | |  |
| Thiacloprid-D4 (IS) |  | 257.1 | 126.0 | 22 | 73.0 | 70 | | 39.1 | 78 | |  |
| Thiamethoxam |  | 292.0 | 211.1 | 8 | 181.1 | 20 | | 132.0 | 24 | |  |
| Thiamethoxam-D4 (IS) |  | 296.1 | 214.2 | 6 | 215.0 | 10 | | - | - | |  |
| **^a^** collision energy | |  |  |  |  |  | |  |  | |  |
|  |  |  |  |  |  |  | |  |  | |  |

**SI Table 5. Recoveries of the tested neonicotinoids at various fortification levels and repeatability.** Honey samples were fortified and analysed in four to five replicates at each fortification level. Measured mean concentrations, standard deviations (SD), relative standard deviations, and recoveries are shown for each analyte at the tested fortification levels. Recoveries were calculated as the percentage of the measured concentration relative to the fortification level.

| **Analyte** | **Fortification levels**  **[µg kg^-1^]** | **n^1^** | **Mean [µg kg^-1^]** | **SD^2^**  **[µg kg^-1^]** | **SD^2^**  **[%]** | **Recoveries [%]** |
| --- | --- | --- | --- | --- | --- | --- |
| Acetamiprid | 0.25 | 5 | 0.27 | 0.01 | 3.1 | 108 |
| Acetamiprid | 0.5 | 5 | 0.5 | 0.01 | 2.7 | 97 |
| Acetamiprid | 1 | 5 | 0.9 | 0.01 | 1.1 | 94 |
| Acetamiprid | 5 | 5 | 4.6 | 0.3 | 6.4 | 91 |
| Acetamiprid | 20 | 5 | 18.9 | 0.3 | 1.5 | 94 |
| Acetamiprid | 50 | 5 | 46.4 | 1.1 | 2.4 | 93 |
|  |  |  |  |  |  |  |
| Clothianidin | 1 | 4 | 1.2 | 0.1 | 7.7 | 118 |
| Clothianidin | 5 | 5 | 4.9 | 0.3 | 5.9 | 98 |
| Clothianidin | 20 | 5 | 19.6 | 0.6 | 3.3 | 98 |
| Clothianidin | 50 | 5 | 47.8 | 1.5 | 3 | 96 |
|  |  |  |  |  |  |  |
| Imidacloprid | 0.5 | 5 | 0.5 | 0.04 | 6.8 | 108 |
| Imidacloprid | 1 | 5 | 1 | 0.03 | 3.2 | 103 |
| Imidacloprid | 5 | 5 | 4.8 | 0.3 | 5.4 | 96 |
| Imidacloprid | 20 | 5 | 19.9 | 0.3 | 1.7 | 99 |
| Imidacloprid | 50 | 5 | 48.1 | 1.1 | 2.4 | 96 |
|  |  |  |  |  |  |  |
| Thiacloprid | 0.25 | 5 | 0.25 | 0.01 | 2.1 | 100 |
| Thiacloprid | 0.5 | 5 | 0.5 | 0.01 | 2.4 | 94 |
| Thiacloprid | 1 | 5 | 0.9 | 0.01 | 0.7 | 94 |
| Thiacloprid | 5 | 5 | 4.6 | 0.3 | 6.5 | 93 |
| Thiacloprid | 20 | 5 | 19.4 | 0.4 | 1.8 | 97 |
| Thiacloprid | 50 | 5 | 47.9 | 0.9 | 1.8 | 96 |
|  |  |  |  |  |  |  |
| Thiamethoxam | 0.25 | 5 | 0.2 | 0.004 | 1.8 | 94 |
| Thiamethoxam | 0.5 | 5 | 0.5 | 0.01 | 2.6 | 92 |
| Thiamethoxam | 1 | 5 | 0.9 | 0.01 | 0.6 | 92 |
| Thiamethoxam | 5 | 5 | 4.6 | 0.4 | 7.9 | 93 |
| Thiamethoxam | 20 | 5 | 19.8 | 0.5 | 2.5 | 99 |
| Thiamethoxam | 50 | 5 | 48.7 | 1.5 | 3 | 97 |

^1^Number of replicates. ^2^Standard deviation.

**SI Table 6. Limit of detection (LOD), Limit of quantification (LOQ) and recovery rates**

| Neonicotinoid | LOD  [μg/kg] | LOQ [μg/kg] | Recoveries  [%]^†^ |
| --- | --- | --- | --- |
| Acetamiprid | 0.05 | 0.25 | 108 |
| Clothianidin | 1 | 1 | 118 |
| Imidacloprid | 0.2 | 0.5 | 108 |
| Thiacloprid | 0.05 | 0.25 | 100 |
| Thiamethoxam | 0.05 | 0.25 | 94 |
|  |  |  |  |

^†^Recoveries at the quantification limits; determined at least four times.

**SI Figure 1. Linearity as visualised with the calibration curves used for the quantitation of the five tested neonicotinoids:** External calibration is shown for **(A)** acetamiprid, **(B)** thiamethoxam, **(C)** thiacloprid, **(D)** imidacloprid and **(E)** clothianidin. Black points show the (seven to eight) calibration levels and the and grey lines show the fitted regression curves. Calibration range, regression equations, and coefficients of determination (R²) are shown for each neonicotinoid.

***1.1 Detailed method of the quantification of neonicotinoid residues in honey:***

*1.1.1 Chemicals & utilities used for the residue analysis with LC-MS* The following reference standards were obtained from LGC Standards GmbH (Wesel, Germany): acetamiprid (C10013000), clothianidin (C11691700), clothianidin-D3 (C11691710), imidacloprid (C14283700, thiamethoxam (C17453000), thiamethoxam-D4 (C17453010). Thiacloprid (37905), thiacloprid-D4 (30673) and acetamiprid-D3 (39246) were purchased from Sigma-Aldrich (Buchs, Switzerland). Imidacloprid-D4 (I274992) was bought from Toronto Research Chemicals (Toronto, Canada).

Acetonitrile SupraSolv (Art. No. 1.00017) and 2-propanol (Art. No, 1.01040), LiChroSolv quality, were purchased from Merck (Darmstadt, Germany). Formic acid solution 50% for HPLC (09676) was acquired from Honeywell Fluka (Buchs, Switzerland) and ammonium formate (70221) was acquired from Supelco (Darmstadt, Germany). For the extraction process of neonicotinoid insecticides, the following substances were used: magnesium sulphate (63136) and sodium hydrogencitrate sesquihydrate (359084) from Sigma-Aldrich (Buchs, Switzerland), sodium chloride (1.06404) and tri-sodium citrate dihydrate (1.06448.0500) from Merck (Darmstadt, Germany), and Bondesil PSA 40 μm (12213024) and Bondesil C18 40 μm (12213012) were from Agilent Technologies (Santa Clara, California, USA). Lastly 1.5 mL Eppendorf Safe-Lock tubes (11.3119.06), 1.5 mL auto-sampler vials (190804.00), 2 mL single-use syringes (3.7410.02), and a single-use polyamide filter (12.3663.01) were obtained from Huberlab (Aesch, Switzerland). Polypropylene (PP) centrifugation tubes (227270) were purchased from Greiner Bio (St. Gallen, Switzerland).

*1.1.2 Honey serving as a blank:* The honey blank used for recovery analyses and serving as a blank was collected in 2023 from colonies of Agroscope in Liebefeld, Switzerland (46.929757°N, 7.424116°E). The honey was tested for the neonicotinoid insecticides acetamiprid, thiamethoxam, clothianidin, imidacloprid and thiacloprid. The tested substances were below LOD, confirming the suitability of this honey as a negative control.

*1.1.3 Extraction of neonicotinoids from honey:* The extraction and analysis of neonicotinoids were carried out employing QuEChERS (quick, easy, cheap, efficient, rugged, safe) extraction, modified from the procedure described by Niell et al., (2014). In brief 1 g of honey was carefully placed into a 50-milliliter polypropylene centrifugation tube and weighed using an analytic balance (Mettler Toledo XP 205, Mettler Toledo, USA, d = 0.0001 g) to correct for deviations in sample weight after analysis. Then, 1 mL of MilliQ-water was added, and the samples were suspended using a Vortex (Vortex-Genie 2, Scientific Industries, USA) and allowed to stand for 15 minutes. Two mL of acetonitrile was added containing the five internal standards acetamiprid-D3, imidiacloprid-D4, thiacloprid-D4, thiamethoxam-D4 and clothianidin-D3 at concentrations of 5 µg/L. Next, the samples were shaken by hand two times for 30 seconds. For the extraction of the neonicotinoids from the aqueous into the acetonitrile phase, a salt mixture consisting of 1.5 g magnesium sulphate (MgSO_4_), 0.3 g sodium chloride (NaCl), 0.2 g tri-sodium citrate dihydrate (C_6_H_5_Na_3_O_7_ · 2H_2_O), and 0.1 g of sodium hydrogencitrate sesquihydrate (HOC(COOH)(CH_2_COONa)_2_ · 1.5H_2_O) was added. Samples were then placed on a sample shaker (UNIMAX 2010, Heidolph, Germany) for ten minutes and subsequently placed in a freezer at -20°C for one hour to freeze out lipid components before centrifugation (Sigma 4-16KS, Sigma, Germany) for 20 minutes at 4°C and 10’000 *g*.

Further purification was achieved by transferring a one mL aliquot into 1.5 mL Safe-Lock tubes containing 30 milligrams of both Bondesil PSA and Bondesil C18. Then aliquots were subjected to two rounds of vortexing for 30 seconds each. Following this, samples were centrifuged at room temperature for 20 minutes at 10’000 *g* using a Centrifuge (5804, Eppendorf, Germany). The resulting supernatant was transferred to a 1.5 mL autosampler through a 45 µm polyamide filter. The purified samples were then subjected to analysis using ultra-high-performance liquid chromatography (UHPLC) combined with mass spectrometry (MS/MS).

*1.1.4 UHPLC‑MS/MS analysis:* Residue analysis was conducted on an Agilent 1290 Infinity II Liquid chromatograph (LC) equipped with an autosampler and coupled to an Agilent 6495C tandem quadrupole mass spectrometer (MS). For chromatographic separation a C18 reverse-phase column (Acquity UPLC HSS T3 Column, 100 Å, 1.8 μm, 2.1 mm × 100 mm, Waters, USA) was used and a volume of 1 µl was injected per sample. The column was kept at a temperature of 40°C and the flow set to 0.5 ml/min. The autosampler was kept at an internal temperature of 10°C. The gradient conditions used for the analysis of the neonicotinoid insecticides in honey can be found in SI Table 2. The mobile phase A (mpA) consisted out of 95 % water + 5 % acetonitrile + 0.01 % formic acid + 5 mM ammonium formate, while the mobile phase B (mpB) was 5 % water + 95 % acetonitrile + 0.01 % formic acid + 5 mM ammonium formate. A needle wash was performed by the autosampler using 2-propanol, acetonitrile and 0.01 % formic acid in water after the analysis of each sample.

Detection and quantification were performed by mass spectrometry (MS) operating in electrospray ionization. The ion source condition is listed in SI Table 3. Qualification and quantitation were based on ion transitions, usually using the transition with the highest peak for quantification and two additional transitions for identification. The qualifiers and quantifiers are listed in SI Table 4.

External calibration based on linear regression (1/x) was performed in MassHunter quantitative software (Version B.08.00, Agilent Switzerland) to compute the concentrations of the target analytes in the samples. Acetamiprid-D3, clothianidin-D3, imidacloprid-D4, thiacloprid-D4 and thiamethoxam-D4 were used as internal standards to correct for variations in sample preparation, ionization efficiency, and instrument response. External calibration based on analyte-to-internal-standard response ratios was used for quantitation of the neonicotinoids. Linearity was assessed over seven to eight calibration levels using linear regression with 1/x weighting.

Recoveries were established using blank honey samples fortified at a minimum of four concentration levels per analyte spanning the validated ranges. Recoveries were assessed in four to five replicate analyses per concentration level, yielding recoveries of 94% to 118% (SI Table 5: Recoveries at various fortification levels and repeatability). The limits of quantification (LOQ) were defined as the lowest concentration level meeting acceptable recovery rates and linearity criteria. The resulting LOQs ranged from 0.25 ng g^-1^ to 1 ng g^-1^ (SI Table 6). Limits of detection (LOD) were determined experimentally by serial dilution of spiked blank extracts until a signal -to-noise ratio of 3:1 was achieved, indicating the lowest concentration level at which the various neonicotinoids could be reliably detected. The LODs ranged from 0.05 ng g^-1^ to 1 ng g^-1^ depending on the neonicotinoid (SI Table 6). Values detected between the LOD and LOQ were set to the LOD for descriptive statistics, whereas values <LOD were treated as non-detects.
